# Clinical Eosinophil-Associated Genes can Serve as a Reliable Predictor of Bladder Urothelial Cancer

**DOI:** 10.3389/fmolb.2022.963455

**Published:** 2022-07-22

**Authors:** Chaojie Xu, Lishan Song, Hui Peng, Yubin Yang, Yi Liu, Dongchen Pei, Jianhua Guo, Nan Liu, Jiabang Liu, Xiaoyong Li, Chen Li, Zhengjun Kang

**Affiliations:** ^1^ The Fifth Affiliated Hospital of Zhengzhou University, Zhengzhou University, Zhengzhou, China; ^2^ Department of Orthopaedics, Affiliated National Hospital of Guangxi Medical University, Nanning, China; ^3^ College of Pharmacy, Shantou University School of Medicine, Shantou, China; ^4^ Department of Biology, Chemistry, Pharmacy, Free University of Berlin, Berlin, Germany

**Keywords:** eosinophil, bladder urothelial carcinoma, WGCNA, risk score, immunotherapy

## Abstract

**Background:** Numerous studies have shown that infiltrating eosinophils play a key role in the tumor progression of bladder urothelial carcinoma (BLCA). However, the roles of eosinophils and associated hub genes in clinical outcomes and immunotherapy are not well known.

**Methods:** BLCA patient data were extracted from the TCGA database. The tumor immune microenvironment (TIME) was revealed by the CIBERSORT algorithm. Candidate modules and hub genes associated with eosinophils were identified by weighted gene co-expression network analysis (WGCNA). The external GEO database was applied to validate the above results. TIME-related genes with prognostic significance were screened by univariate Cox regression analysis, lasso regression, and multivariate Cox regression analysis. The patient’s risk score (RS) was calculated and divided subjects into high-risk group (HRG) and low-risk group (LRG). The nomogram was developed based on the risk signature. Models were validated *via* receiver operating characteristic (ROC) curves and calibration curves. Differences between HRG and LRG in clinical features and tumor mutational burden (TMB) were compared. The Immune Phenomenon Score (IPS) was calculated to estimate the immunotherapeutic significance of RS. Half-maximal inhibitory concentrations (IC50s) of chemotherapeutic drugs were predicted by the pRRophetic algorithm.

**Results:** 313 eosinophil-related genes were identified by WGCNA. Subsequently, a risk signature containing 9 eosinophil-related genes (*AGXT, B3GALT2, CCDC62, CLEC1B, CLEC2D, CYP19A1, DNM3, SLC5A9, SLC26A8*) was finally developed *via* multiplex analysis and screening. Age (*p* < 0.001), grade (*p* < 0.001), and RS (*p* < 0.001) were independent predictors of survival in BLCA patients. Based on the calibration curve, our risk signature nomogram was confirmed as a good predictor of BLCA patients’ prognosis at 1, 3, and 5 years. The association analysis of RS and immunotherapy indicated that low-risk patients were more credible for novel immune checkpoint inhibitors (ICI) immunotherapy. The chemotherapeutic drug model suggests that RS has an effect on the drug sensitivity of patients.

**Conclusions:** In conclusion, the eosinophil-based RS can be used as a reliable clinical predictor and provide insights into the precise treatment of BLCA.

## Introduction

Bladder urothelial carcinoma (BLCA) is a malignancy of the bladder uroepithelium with approximately 500,000 newly diagnosed cases and 200,000 deaths worldwide each year (Siegel et al., 2022). A quarter of BLCA cases are muscle-invasive bladder cancer (MIBC) by statistics (Robertson et al., 2017), while non-muscle invasive bladder cancer (NMIBC) has a fast progression and a high recurrence rate (Sylvester et al., 2016). The recurrence probability of NMIBC patients undergoing transurethral resection and combination therapy is as high as 50–70% (Robertson et al., 2018). However, MIBC often has a poor prognosis due to early occult metastatic spread, and its 5-years survival rate does not exceed 50% (Hurst and Knowles, 2022). Despite multiple therapies, overall survival and prognosis for BLCA remain poor severely. Therefore, emerging biomarkers are urgently identified for the clinic.

Increased eosinophil counts are commonly seen in various malignancies (Samoszuk, 1997), it was reported that eosinophils in the peripheral blood of cancer patients increased compared with normal people (Olsen and Spry, 1985). Eosinophils are generally considered granulocytes involved in allergic disease responses and parasitic infection responses. Eosinophilic tumor infiltration occurs during the proliferation of certain types of tumors, such as breast cancer and gastric adenocarcinoma (Costello et al., 2005). Eosinophils readily respond to a variety of stimuli by synthesizing and secreting a large number of molecules, including unique granulin proteins that may kill tumor cells (Grisaru-Tal et al., 2020). Studies have shown that some cytokines (IL-4, IL-5) can increase the anti-tumor activity of eosinophils (Furbert-Harris et al., 2003).

Immunotherapy made tremendous progress and has been an indispensable component in the treatment of advanced solid tumors in the past decade. Cancer immunotherapy is not limited to understanding the relationship between cancer and the immune system, and immunocytes have also been used to predict the prognosis of cancer patients (Havel et al., 2019; Kruger et al., 2019). Immune checkpoint inhibitors (ICIs) have been formally used in BLCA in 2016 (Özdemir et al., 2018), moreover, immunotherapy targeting immune checkpoints is still based on PD-L1 inhibitors (Rhea et al., 2021). The safety and efficacy of immunotherapy for BLCA have long been proven relative to other second-line treatments in clinical trials (Bellmunt et al., 2017). T cells, macrophages, and tumor-associated fibroblasts, myeloid-derived suppressor cells in the microenvironment, almost significantly inhibit effective immunotherapy (Hessmann et al., 2020). Among them, eosinophil and eosinophil-associated signaling pathways play a key role in antitumor immunity and induction of epithelial mesenchymal transition (De Palma et al., 2017; Korbecki et al., 2020). Therefore, the strategy of autoimmune profiling may be the most reliable and promising strategy to comprehensively assess tumor sensitivity to clinical therapy. However, the survival value of eosinophils in BLCA patients and the biological role of the tumor immune microenvironment (TIME) are still not evident.

We investigated the potential role of eosinophils in BLCA TIME with the TCGA-BLCA and GSE31684 cohorts. Eosinophil analysis was obtained using the CIBERSORT algorithm, and significant modules related to eosinophils were identified by weighted gene co-expression network analysis (WGCNA). We established a nomogram combining risk signature and clinical features according to eosinophil-associated genes. In addition, we also verified the synergistic effect of risk score (RS) and tumor mutational burden (TMB), the relationship of immunotherapy responsiveness and drug sensitivity.

## Methods

### Data Collection

Transcriptome data and clinical annotations were downloaded from the TCGA-BLCA (The Cancer Genome Atlas-Bladder urothelial carcinoma) cohort consisting of 433 RNA-sequencing samples, including 19 normal samples and 414 tumor samples. After removing the sample without clinical follow-up information from the TCGA-BLCA cohort, 404 cases were applied for the following analysis. The four types of somatic mutation data in BLCA patients were also obtained from the TCGA database. Furthermore, we took the GSE31684 cohort data from the GEO database for external validation.

### The Landscape of Immune Cell Infiltration

Sequencing data from computational samples were analyzed *via* the CIBERSORT algorithm to obtain the abundance of 22 tumor-infiltrating immune cell (TIC) subtypes. The cellularity of the tumor immune microenvironment were characterized by these subtypes (Langfelder and Horvath, 2008).

### Weighted Gene Co-Expression Network Analysis

The 16,394 gene matrix from the TCGA-BLCA cohort was imported into the CIBERSORT. The soft threshold power (*β*) from 1 to 20 was selected as a candidate, and the corresponding power values were calculated using the pickSoftThreshold function, the best power value was selected to build the proximity matrix, and our gene distribution was made to conform to the scale-free network according to the connectivity. Using the TOM matrix obtained from gene expression, the genes were again continued to be clustered, the minimum number of module genes was set, and the gene clustering results were cut to obtain different gene modules. The “dynamic tree cutting” algorithm was used to introduce similar genes into the same candidate module. In addition, to realize the correlation analysis between the module eigengenes and the sample traits, we performed the Pearson correlation test was performed. Finally, we focused on the “eosinophils” population and extracted the modules most significantly associated with eosinophils for subsequent analysis.

### Construction of Eosinophil-Related Prognostic Features

The 313 genes from the most important module were taken for further screening to discuss the predictive effects of eosinophil-related genes. First, candidate genes significantly associated with overall survival were screened out by univariate Cox regression analysis (*p* < 0.05). Then the range of candidate genes is further narrowed by LASSO algorithm. Finally, 9 genes associated with overall survival were finally screened out by multivariate Cox regression. A prognostic risk model including these 9 eosinophil-related genes was established.

RS was calculated according to the expression of differential genes and regression analysis coefficient values. The formula is shown below:

$$riskscore=\overset{n}{\underset{i=1}{\sum }}\left(coefi*Xi\right)$$

Here, *β* is the regression coefficient in the multivariate Cox regression analysis (Lossos et al., 2004).

### Validation of Prognostic Eosinophil-Related Features

According to the above formula, there is one RS for each BLCA sample. All BLCA samples were divided into two subgroups, high-risk group (HRG) and low-risk group (LRG), using the median of RS as the cutoff value. A K-M survival curve was drawn to compare the prognostic differences between the two subgroups, and this process was performed in the “survival” package in R. In addition, transient receiver operating characteristic (ROC) curves were plotted to assess performance. Then, univariate and multivariate Cox regression analysis was performed to confirm the RS as a meaningful independent influencing factor for BLCA patients. The correlations of RS with clinical variables were visualized by the “pheatmap” package.

### Gene Set Enrichment Analysis

The c2.cp.kegg.v7.4.symbols collection were used to explore the function annotation in HRG and LRG by GSEA software. Results with *p* value < 0.05 were being considered statistically significant. The first eight results were selected for visualization.

### Establishment and Verification of the Nomogram

ROC analysis of gender, age, RS, tumor grade, and clinicopathological stage was performed to determine the best prognostic indicators for 1, 3, and 5-years overall survival (OS) in patients (Blanche et al., 2013). To provide a quantifiable tool to predict 1-, 3-, and 5-years OS, the nomogram integrating risk signature and other clinicopathological features were constructed. Finally, the calibration curves were drawn to verify the performance of the nomogram.

### Correlation Between Tumor Mutational Burden and Risk Score

Somatic mutation data from the TCGA-BLCA cohort were obtained from the TCGA database. TMB refers to the number of somatic non-synonymous mutations in a specific genomic region, which can indirectly reflect the ability and degree of tumor production of neoantigens, and predict the immunotherapy efficacy of various tumors. The “maftools” R package (Yoshihara et al., 2013) was used to detect the number of somatic non-synonymous point mutations in each sample.

### Correlation of Risk Score With Tumor Immune Microenvironment Characterization

Seven methods were applied to assess immune cell infiltration in the tumor microenvironment, including XCELL, TIMER, QUANTISEQ, MCPcounter, EPIC, CIBERSORT, and CIBERSORT-ABS. ESTIMATE algorithm, which can be based on gene expression data, estimates the stromal score and immune score of a tumor sample for representing the presence of stromal and immune cells. The two scores are summed to obtain the ESTIMATE score, which can be used to estimate tumor purity. Correlation between RS and TICs was performed using Spearman correlation analysis.

### Gene Set Variation Analysis

We downloaded hallmark gene sets from the MSigDB database (Chan et al., 2019). To assess relative pathway activity in individual samples, the applied gene set variation analysis (GSVA) (Rizvi et al., 2015) in the GSVA package was used to assign pathway activity estimates.

### Predict Patient Response to Immunotherapy

Immune checkpoints have been defined as key targets for the inhibition of immune cell function. In this study, we analyzed the expression levels of 47 immune checkpoint blockage-related genes in HRG and LRG. Immunophenoscore (IPS) determines the immunogenicity of a tumor and predicts the response to immune checkpoint inhibitor therapy. IPS calculates scores for each of the four different immunophenotypes (antigen-presenting, effector, suppressor, and checkpoint), and the IPS z-score is an integration of all four, and the higher the IPS z-score, the more immunogenic the sample (Charoentong et al., 2017).

### Predicting Chemotherapy Effect

To investigate the correlation of RS with chemotherapeutic drug sensitivity, we used the R package “pRRophetic” to appraise the half-maximal inhibitory concentration (IC50) of BLCA samples between HRG and LRG. The package “pRRophetic” can estimate the IC50 of chemotherapeutic drugs by constructing a ridge regression model based on Genomics of Cancer Drug Sensitivity (GDSC) (www.cancerrxgene.org/) cell line expression profiles and TCGA gene expression profiles (Rosenberg et al., 2016).

### Statistical Analysis

The Wilcoxon test was used to compare two groups, and the Kruskal–Wallis test was used to compare more than two groups. The Kaplan-Meier log-rank test was used to analyze the survival curves. Associations between RS subgroups and somatic mutational burden were analyzed with the chi-square test, and Spearman analysis was used to calculate correlation coefficients. Results were further analyzed using the CIBERSORT algorithm (*p* < 0.05). Two-sided *p* < 0.05 was considered statistically significant. All statistical analyses were performed using R software (version 4.1.1).

## Results

### Landscape of Tumor Immune Microenvironment in Bladder Urothelial Carcinoma

**FIGURE 1:**
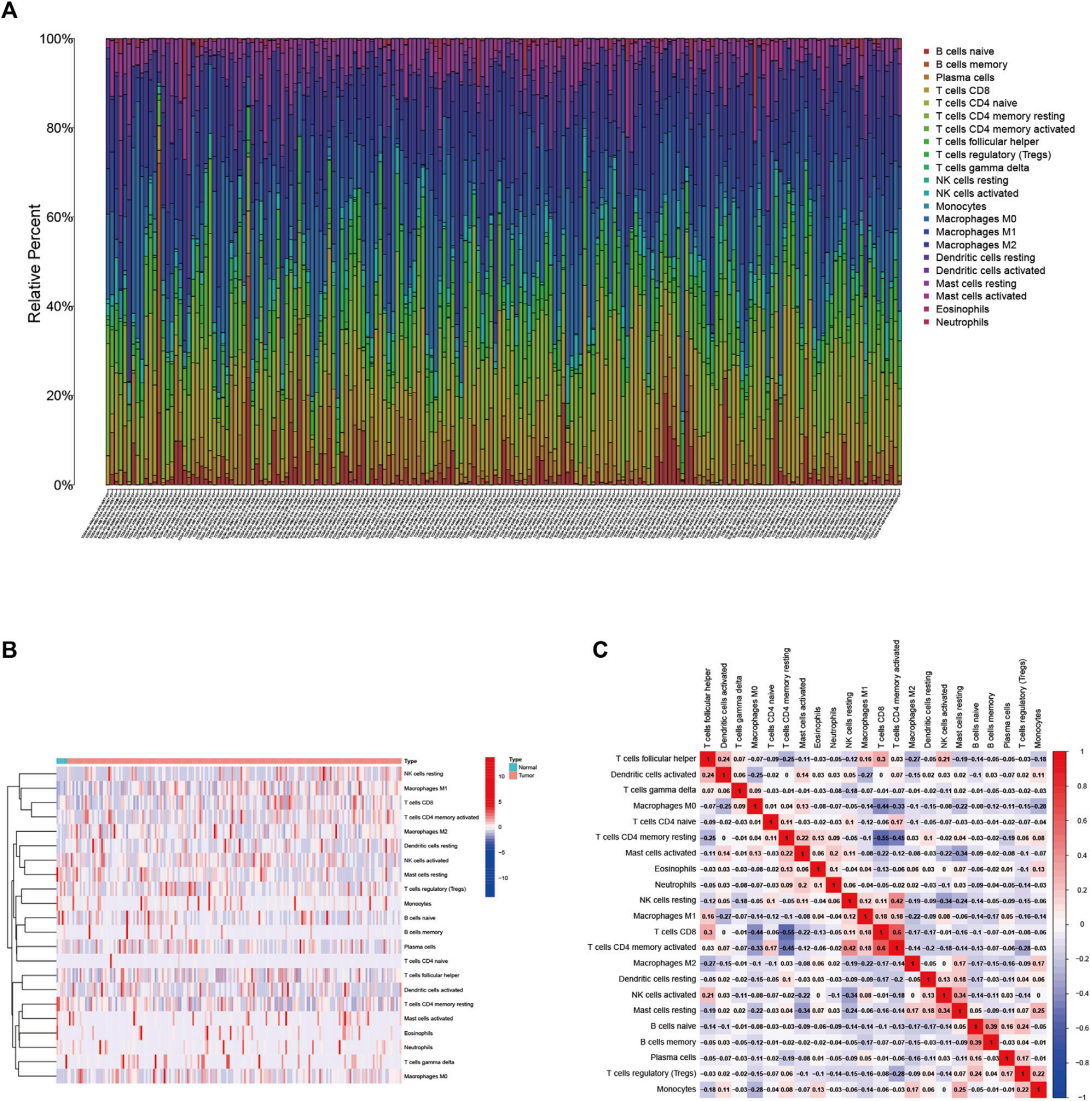
**(A)** Immune cell infiltration in the BLCA tumor immune environment. Subpopulations of 22 immune cell subtypes **(B)** and scale heatmap of 22 TICs in each BLCA sample **(C)**. Intrinsic correlation of 22 infiltrating immune cells in EBLCA.

We employed the CIBERSORT algorithm (Supplementary Table S1) to clarify the synthesis of TIME. The abundance of the 22 TIC types is shown in Figure 1A. To explore and characterize the relationship between TIME patterns and clinical phenotypes, we created a comprehensive heatmap (Figure 1B). We visualized the integrated landscape of TIME to show the correlation between immune cells (Figure 1C), and found that T cells CD4 memory activated and T cells CD8 have the largest positive correlation (r = 0.6, *p* < 0.05), while T cells CD8 and T cells CD4 memory resting with the largest negative correlation (r = −0.55, *p* < 0.05). The largest positive correlation with Eosinophils was T cells CD4 memory resting and Monocytes (r = 0.13, *p* < 0.05), and the largest negative correlation was T cells CD8 (r = −0.13, *p* < 0.05).

### Establishment of the Weighted Gene Co-Expression Network Analysis Network

**FIGURE 2:**
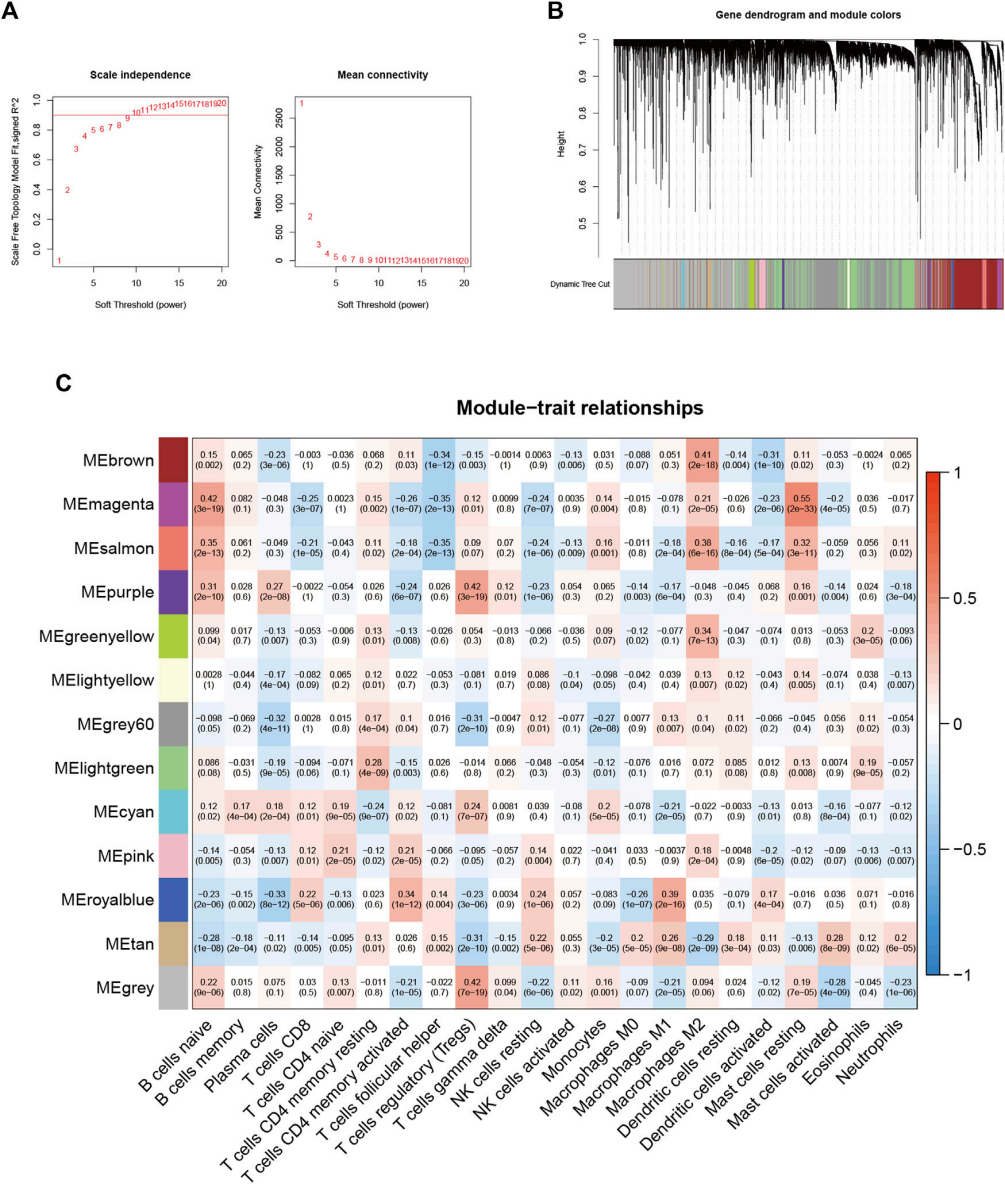
Choosing an appropriate soft threshold (power) and building a hierarchical clustering tree **(A)** The choice of the soft threshold enables the scale-free topology to achieve an exponent of 0.90, and the average connectivity for 1–20 soft threshold powers is analyzed **(B)** M2 macrophage-related genes with similar expression patterns were merged into the same module using a dynamic tree-cutting algorithm, creating a hierarchical clustering tree. Heatmap of correlations between **(C)** modules and immune-infiltrating cells (traits).

Based on the 16,394 genes as well as immune-infiltrating subsets, we construct the scale-free network by defining the first power value when the scale-free topology index reaches 0.90, which is 9 (Figure 2A) and setting it as the optimal soft threshold power (*β*). The similarly expressed pattern genes were introduced into the same module through a dynamic tree-cutting algorithm (module size = 60) to form a hierarchical clustering tree with different modules. Hierarchical cluster analysis was performed based on weighted correlations and the clustering results were segmented according to the set criteria to obtain 13 gene modules (Figure 2B). Each column of Figure 2C represents a TIC type, and each row represents candidate modules with trait carrier genes. The green-yellow module is highly correlated with eosinophils (r = 0.2, *p* < 0.01) in Figure 2C, the genes in this module (Supplementary Table S2) were applied for further studies.

### Development of Risk Signatures

**FIGURE 3:**
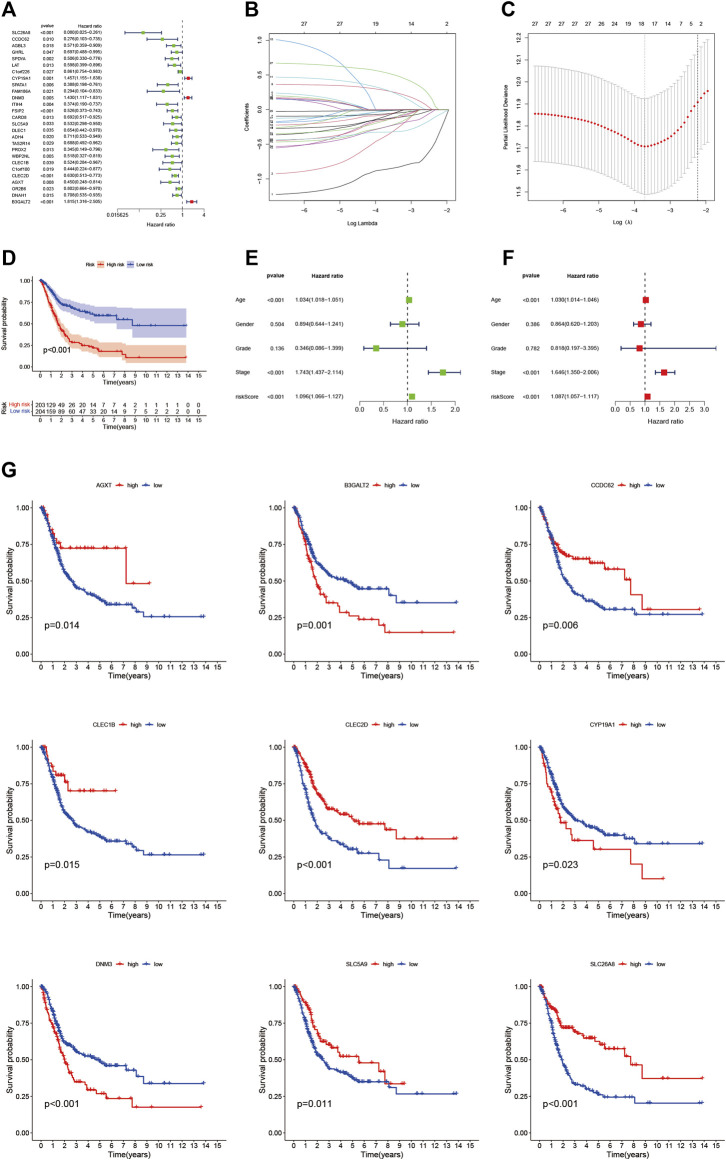
**(A)** Nomogram between 27 eosinophil-related genes and overall survival screened by univariate Cox regression analysis **(B)** LASSO coefficient profiles of 22 candidate genes. A vertical line is drawn at the value chosen by 10-fold cross-validation **(C)** Ten-fold cross-validation for tuning parameter selection in lasso regression. Vertical lines are drawn from the best data according to the minimum criterion and 1 standard error criterion. The vertical lines on the left represent the final 9 genes identified **(D,G)** Kaplan-Meier curve analysis showing the difference in overall survival between high-risk and low-risk groups **(E)** Univariate Cox regression results for overall survival **(F)** Multivariate Cox regression results for overall survival.

Twenty-seven eosinophil-related genes were screened by univariate Cox regression analysis (*p* < 0.05, Supplementary Table S3), and a forestogram was drawn (Figure 3A). To avoid overfitting, we performed Lasso regression on these 27 genes and identified 18 eosinophil-related genes (Figure 3B), furthermore determining the optimal value of the penalty parameter by cross-validation (Figure 3C). We hence identified 9 eosinophil-related genes (*AGXT, B3GALT2, CCDC62, CLEC1B, CLEC2D, CYP19A1, DNM3, SLC5A9, SLC26A8*) as hub genes by multivariate Cox regression. All nine genes were considered beneficial prognostic indicators (Supplementary Table S4).

More importantly, nine hub genes were incorporated into the risk profile of BLCA patients. The RS was computed:

$$\begin{array}{l} risk\;score\;\left(RS\right)=-\left(1.5416\times SLC26A8\right)-\left(0.9195\times CCDC62\right)+\left(0.3704\times CYP19A1\right)+\left(0.3902\times DNM3\right)-\left(0.6255\times SLC5A9\right)\\ -\left(0.4651\times CLEC1B\right)-\left(0.4201\times CLEC2D\right)-\left(0.5352\times AGXT\right)+\left(0.6509\times B3GALT2\right) \end{array}$$

Finally, BLCA samples were divided into HRG and LRG according to the Bit RS.

### Validation of Risk Prognostic Features

**FIGURE 4:**
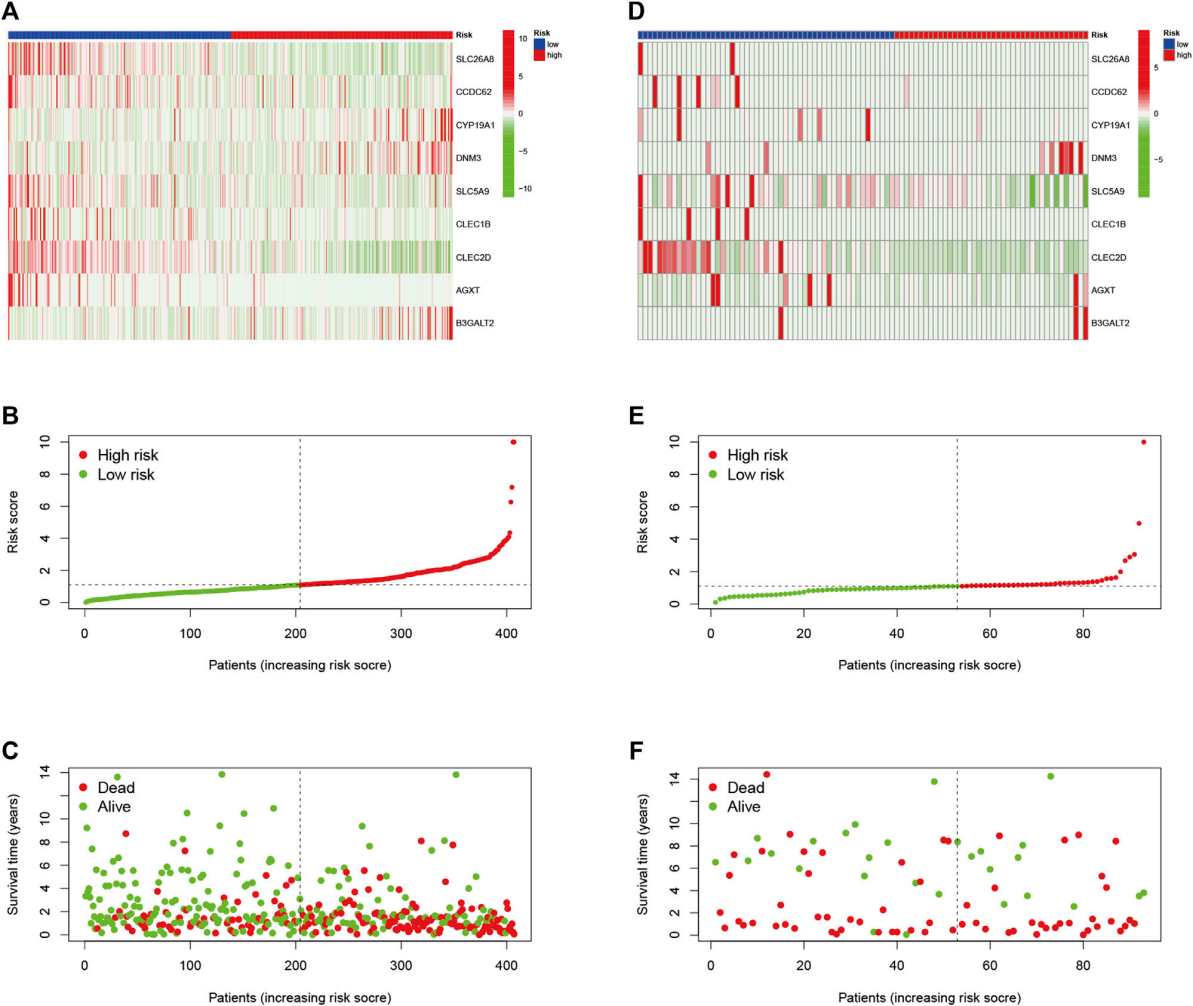
**(A)** Confirmation of prognostic risk scores in the TCGA cohort **(B)** Polygenic model risk score distribution in the TCGA cohort **(C)** Survival status and duration of BLCA patients in the TCGA cohort **(D)** Confirmation of prognostic risk scores in the GEO cohort **(E)** Polygenic model risk score distribution in the GEO cohort **(F)** Survival status and duration of BLCA patients in the GEO cohort.

We found the OS time of HRG patients in the TCGA cohort is significantly shorter than that of LRG patients through the K-M survival curve (*p* < 0.001, Figure 3D), which proved that our RS has a good performance. There was also a difference in OS between the two groups of patients with high and low hub gene expression (Figure 3G). In addition, we showed RS in the TCGA cohort (Figure 4A), polygenic model RS distribution (Figure 4B), survival status, and longevity of BLCA patients (Figure 4C). By univariate Cox regression analysis, we obtained a hazard ratio (HR) of 1.096 for RS (95% CI 1.066–1.127, Figure 3E). The results of multivariate Cox regression analysis (HR = 1.087, 95% CI 1.057–1.117, Figure 3F) supported RS as an independent prognostic indicator in BLCA. The above results clarified these 9 hub genes have a good prediction of clinical outcomes. More importantly, this feature was validated by an external database GSE31684. The outcomes showed the distribution of nine gene expression patterns, sample prognostic information, and RS in the external validation cohort (Figures 4D–F). These results suggested that this feature has a stable and robust prognostic value.

### Functional Analysis of Eosinophil-Related Genes

**FIGURE 5:**
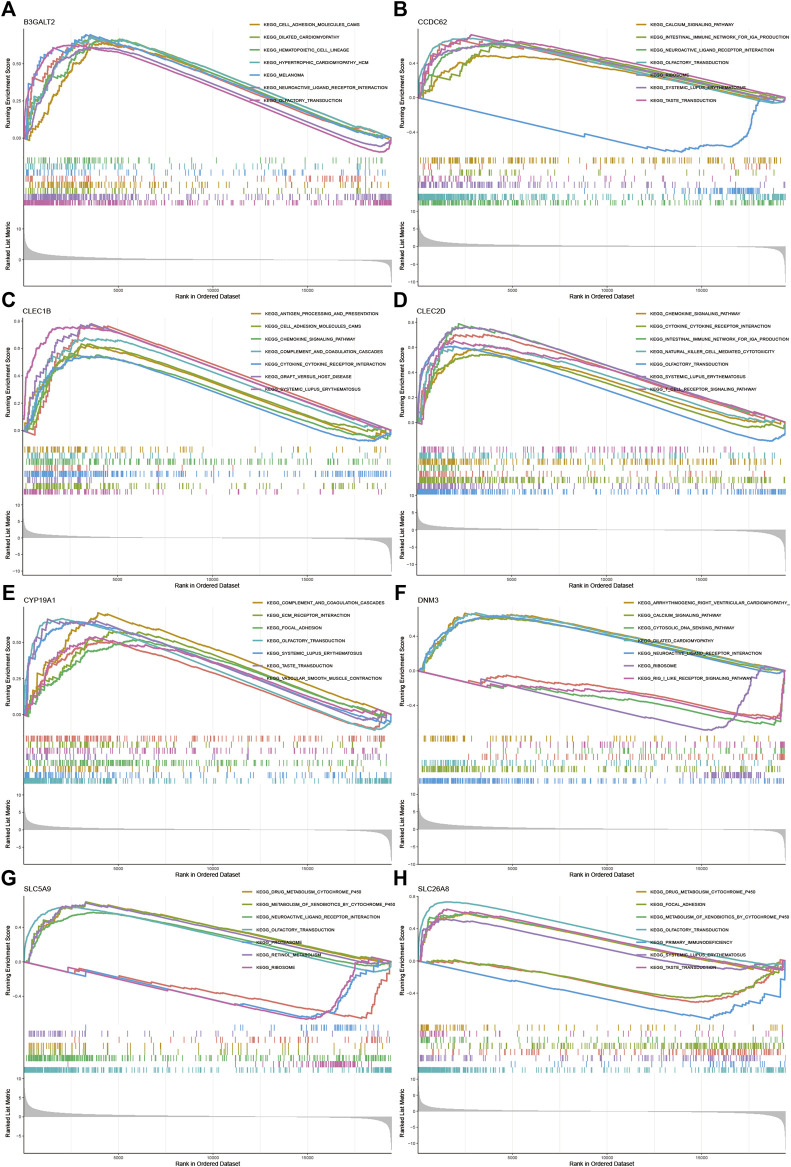
GSEA for samples with high and low expression of 9 central genes **(A)** Enriched gene set collected in KEGG for samples with high *B3GALT2* expression **(B)** Enriched gene set collected in KEGG for samples with high *CCDC62* expression **(C)** Enriched gene set collected in KEGG for samples with high *CLEC1B* expression **(D)** Enriched gene set collected in KEGG for samples with high *CLEC2D* expression **(E)** Enriched gene set collected in KEGG for samples with high *CYP19A1* expression **(F)** High *DNM3* expression sample enriched gene set collected in KEGG **(G)** Enriched gene set collected in KEGG for samples with high *SLC5A9* expression **(H)** Enriched gene set in the KEGG collection for samples with high *SLC26A8* expression.

All samples were divided into low and high expression groups by the median expression of the hub genes that served as the cut-off point. Functional enrichment of high and low hub gene expression was identified by GSEA subsequently. The KEGG enrichment analysis showed that the high expression of DNM3 was related to signaling pathways such as arrhythmogenic right ventricular cardiomyopathy and neuroactive ligand-receptor interaction; the high expression of *SLC5A9* was related to signaling pathways such as drug metabolism—cytochrome p450 and neuroactive ligand-receptor interaction; the high expression of *SLC26A8* was related to olfactory transduction and drug metabolism—cytochrome p450 and other signaling pathways (Figure 5).

### Association of Risk Characteristics With Clinicopathological Variables

**FIGURE 6:**
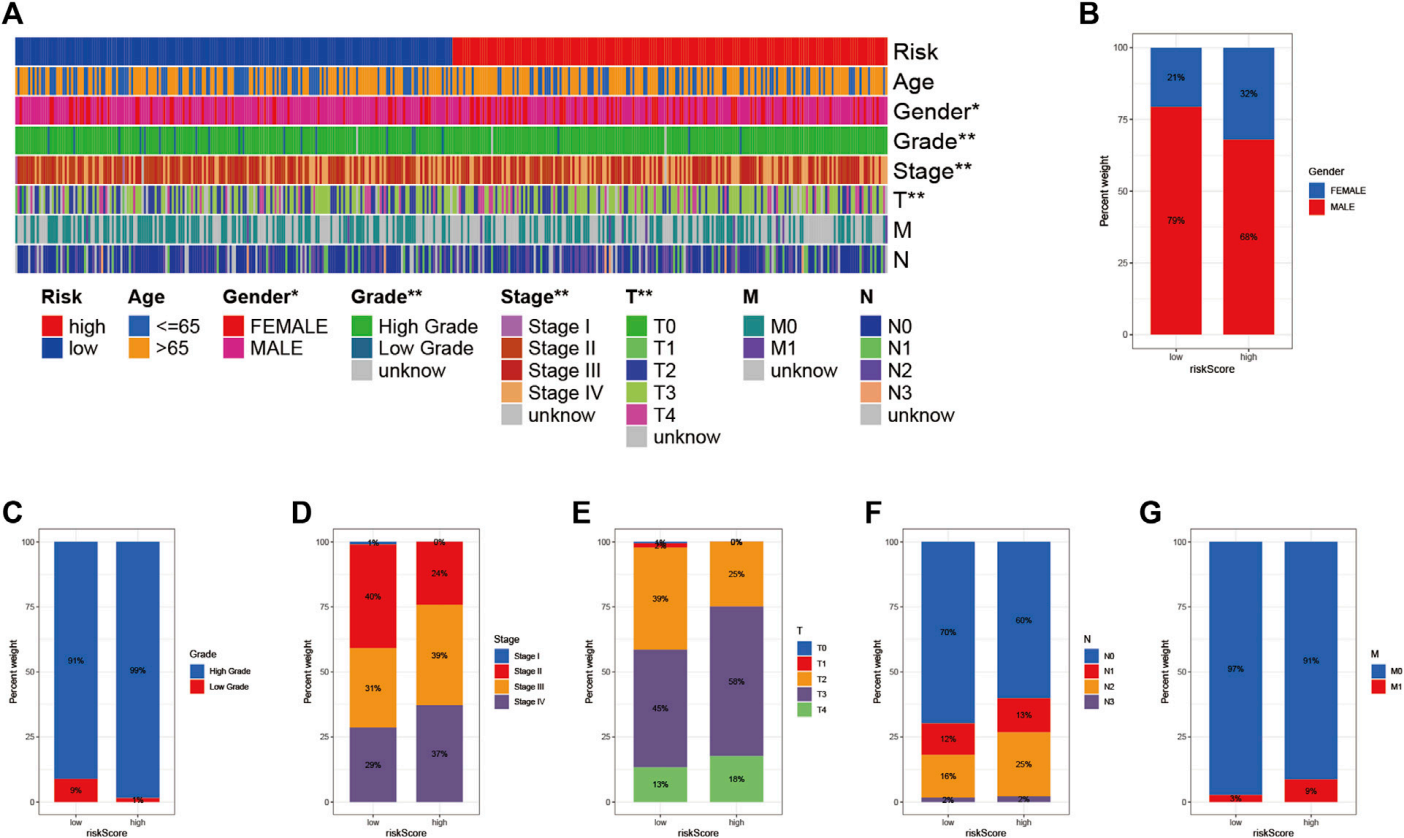
Clinical significance of prognostic risk characteristics **(A)** heatmap showing the distribution of clinical characteristics and corresponding risk scores in each sample. Incidence of clinical variable subtypes of LRG/HRG **(B)** Gender **(C)** Grade **(D)** Stage **(E)** Stage T **(F)** Stage N, and **(G)** Stage M.

To visualize the distribution of clinical variables in the LRG/HRG subgroup, we plotted Figure 6A. Clinical subtype scores between HRG and LRG based on sex, tumor grade, clinical stage, T, N, and M stage are shown in Figures 6B–G.

### Construction of Prognostic Nomogram

**FIGURE 7:**
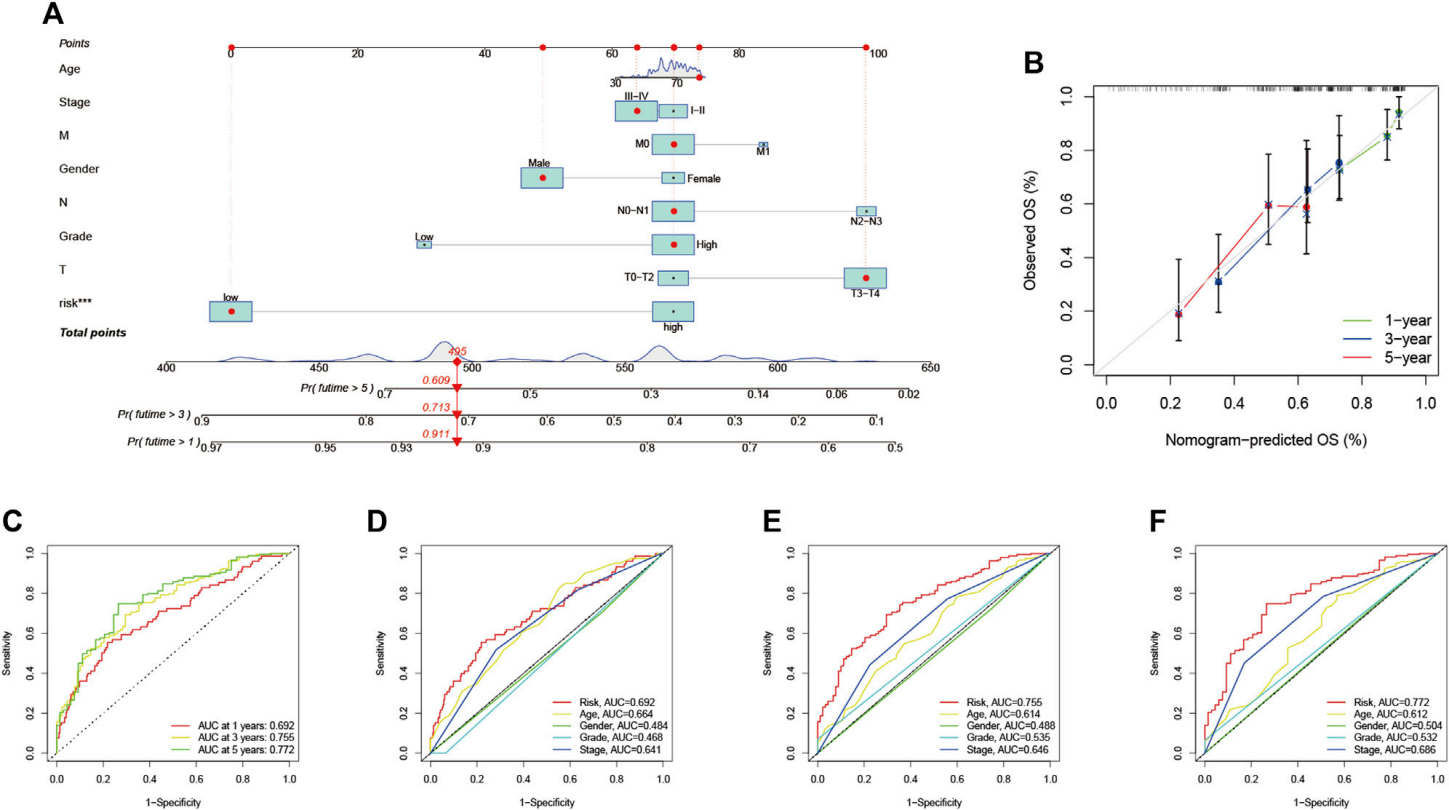
Validation of prognostic efficiency of risk signatures **(A)** Nomogram was used to predict survival in BLCA patients **(B)** 1-, 3-, and 5-years nomogram calibration curves **(C)** ROC analysis was used to estimate the predictive value of prognostic features. The area under the **(D–F)** curve (AUC) of the risk score for predicting overall survival at 1, 3, and 5 years and other clinical characteristics.

The ROC curves indicated a high prognostic validity, with AUC values of 0.692, 0.755, and 0.772 for 1-, 3-, and 5-years OS, respectively (Figure 7C). Moreover, we combined gender, tumor grade, T, N, and M stages for AUC analysis of 1-year (Figure 7D), 3-years (Figure 7E), and 5-years (Figure 7F) OS to further validate that RS had the best prognostic value among multiple clinicopathological variables. The results elucidated that RS obtained the highest AUC value. Then, we developed a prognostic nomogram composed of RS, gender, tumor grade, clinical stage, T, N, and M stage for quantitative prognostic prediction (Figure 7A). The survival rate of BLCA patients can be accurately predicted based on nomogram. For example, an 80-year-old male was diagnosed with bladder cancer. The patient’s clinical information was T3N1M0 with stage III and had a low RS. We could then calculate the patient’s total score as 495, corresponding to survival rates of 0.911, 0.713, and 0.609 for 1, 3, and 5 years, respectively. Finally, the calibration curve was established to evaluate the predictive performance of the nomogram, which showed good prognostic prediction performance (Figure 7B).

### Association of Risk Signature With Tumor Mutational Burden

**FIGURE 8:**
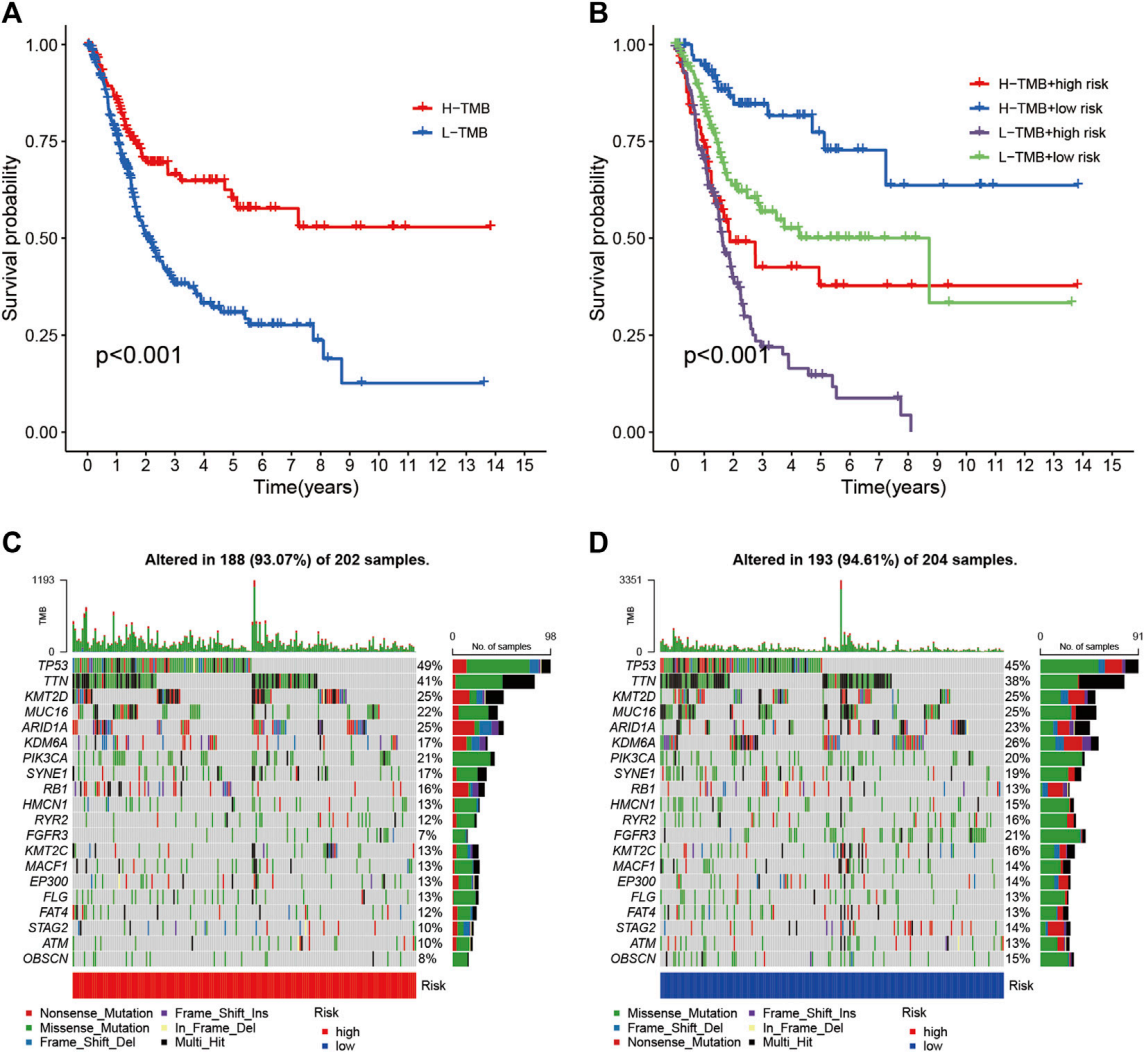
Correlation between risk score and TMB **(A)** Kaplan-Meier curves of high TMB and low TMB groups **(B)** Kaplan-Meier curve stratification of patients according to TMB and risk signature. The oncoPrint was constructed using high risk score **(C)** and low risk score **(D)**.

To reveal the relationship between RS and TMB and to reveal the genetic variation of RS subtypes, we plotted K-M survival curves based on TBM levels, indicating that high TMB values led to longer OS (*p* < 0.001, Figure 8A). We divided the patients into four subgroups according to the median of RS and TMB. As shown in Figure 8B, the RS subgroup exhibited significant prognostic differences with the low and high TMB subgroup, and TMB levels did not interfere with the RS prognostic prediction performance (*p* < 0.001). These results further confirm that RS may serve as an independent prognostic predictor and has the potential to evaluate the clinical outcomes of antitumor immunotherapy.

To explore the relationship between RS and gene mutations, we analyzed the distribution of gene mutations between HRG and LRG and mapped the top 20 gene waterfalls with the most frequent somatic mutations between HRG and LRG (Figures 8C,D). Significantly, mutated gene (SMG) mutation profiles indicated that *TP53* (49 vs. 45%) experienced a higher rate of somatic mutation in HRG core subtypes, while *FGFR3* (21 vs. 7%) had a higher rate of LRG somatic mutation rate. These findings may contribute to new insights into the intrinsic link between eosinophil infiltration and somatic variation in BLCA immunotherapy.

### Risk Signature in Tumor Immune Microenvironment Context

**FIGURE 9:**
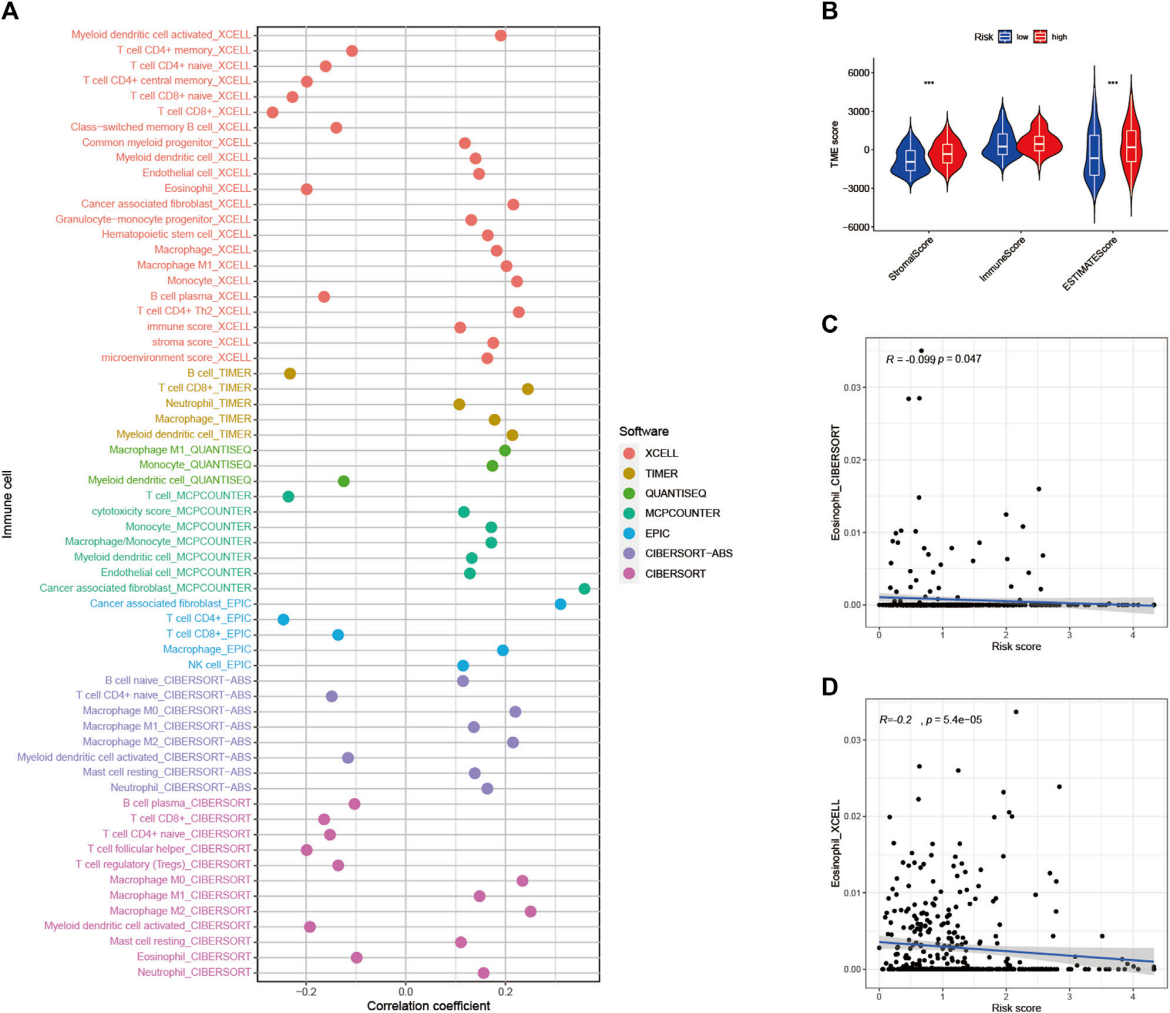
Estimated abundance of tumor-infiltrating cells. Patients in the **(A)** high-risk group had a stronger correlation with tumor-infiltrating immune cells, as shown by the Spearman correlation analysis **(B)** Association between prognostic risk signatures and central immune checkpoint genes. The correlations predicted by the two methods CIBERSORT **(C)** and XCELL **(D)** were validated.

To explore the role of RS and TIME, we analyzed the association of RS with immune infiltration by Spearman correlation (Figure 9A, Supplementary Table S5). The ESTIMATE analysis showed a significant upward trend in HRG matrix scores. Furthermore, ESTIMATE scores were significantly upregulated in higher-risk samples (Figure 9B). Validation of the correlation predicted by the two methods CIBERSORT (Figure 9C) and XCELL (Figure 9D) showed that our analysis is dependable.

### Enrichment of Signaling Pathways in High-Risk Group/Low-Risk Group

**FIGURE 10:**
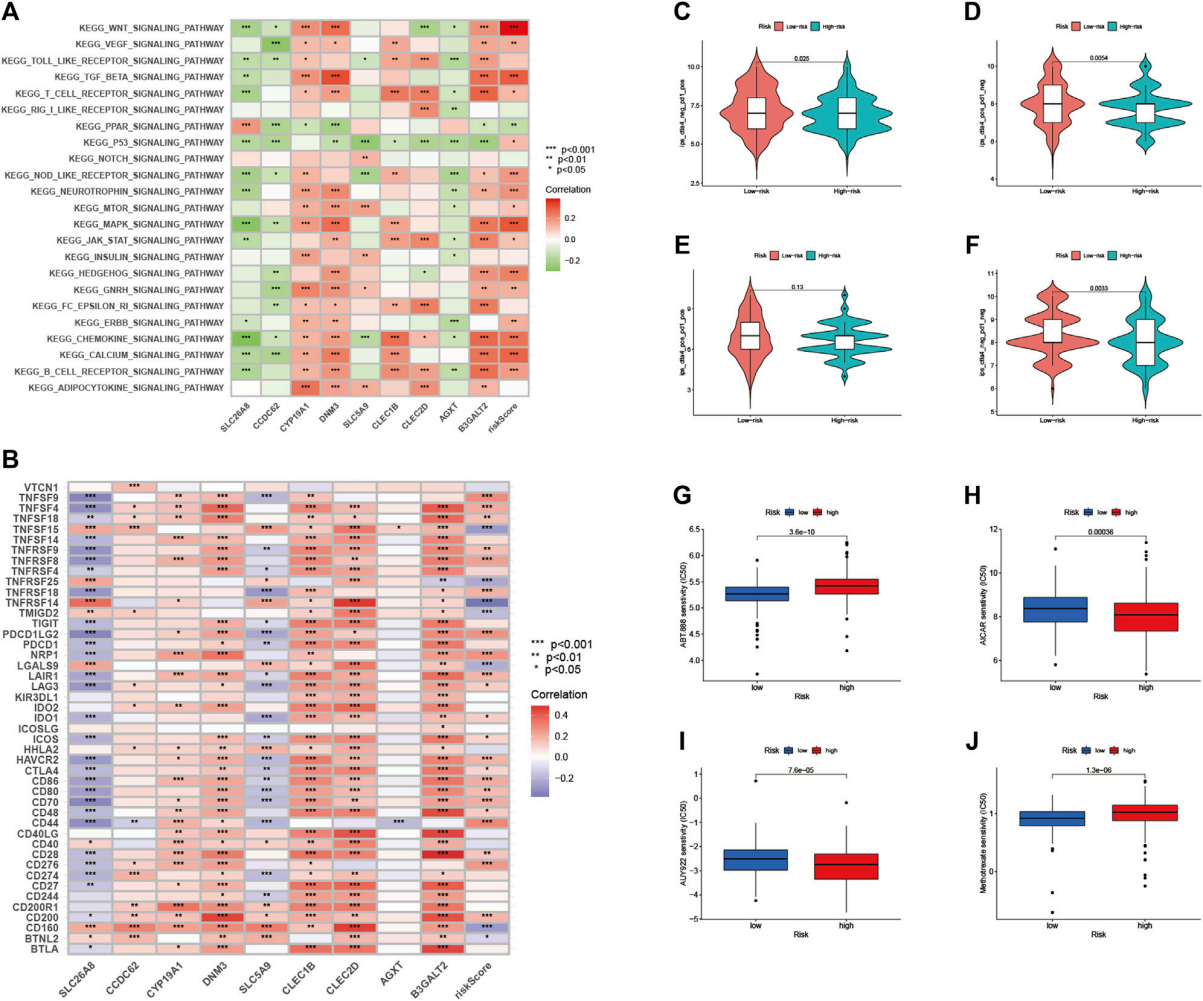
Enrichment pathways for GSVA **(A)** heatmap showing the correlation of representative pathway items on KEGG with risk scores. Predicting immunotherapy response **(B)** Association of immune checkpoint blockade gene expression levels with risk scores **(C–F)** IPS score distribution map. Estimates of chemotherapy effect risk scores. Sensitivity analysis of **(G)** Veliparib in patients with high and low risk scores. Sensitivity analysis of **(H)** cisplatin in patients with high and low risk scores **(I)** Sensitivity analysis of paclitaxel in patients with high and low risk scores **(J)** Sensitivity analysis of methotrexate in patients with high and low risk scores.

As shown in Figure 10A, we performed gene set variation analysis (GSVA) to further reveal the biological roles of different risk groups in tumorigenicity and progression. Subjects in the LRG showed enhanced *KEGG/PPAR* pathway activity. Most genes with high expression levels in the HRG were enriched in *KEGG/WNT*, *KEGG/TGF/BETA*, *KEGG/MAPK*, and *KEGG/CHEMOKINE* signaling pathway.

### Immunotherapy Prediction

Since there is no data information for immunotherapy in the TCGA-BLCA dataset, we indirectly analyzed the response to immunotherapy. As shown in Figure 10B, most immune checkpoint blockade-related genes (*TNFSF9, TNFSF4, TNFRSF8*) were significantly positively associated with RS.

In this risk scoring system, patients in HRG have a higher IPS score at the time of immunotherapy (Figure 10C: PD1-positive and CTLA4-negative; Figure 10D: PD1-negative and CTLA4-positive). It is easy to draw from Figure 10F that LRG is more suitable for novel immunotherapy (Figure 10F: PD1-negative and CTLA4-negative). These results confirmed that RS is associated with immunotherapy.

### Prediction of Response to Chemotherapy

We estimated the IC50s of 4 chemotherapeutic agents (Veliparib, paclitaxel, cisplatin, and methotrexate) in BLCA patients. Veliparib and methotrexate exhibited higher IC50s in HRG signature patients (all *p* < 0.001; Figures 10G,J). In contrast, paclitaxel and cisplatin exhibited higher IC50s in LRG patients (all *p* < 0.001; Figures 10H,I). These results suggested that RS effects the drug sensitivity of patients.

## Discussion

As the ninth most common cancer in the world, BLCA with high recurrence and high invasiveness poses a great threat to the health of all human beings (Cumberbatch et al., 2018). Cisplatin-based chemotherapy is the universal treatment for locally advanced or metastatic BLCA in the clinic currently (von der Maase et al., 2005), however, the efficacy is usually unsatisfactory due to the physical condition of the patients and various adverse reactions (Chen et al., 2021). In recent years, immunotherapy has developed rapidly, with a variety of ICIs appearing and being used to treat advanced BLCA (Chism, 2017). Although the safety and efficacy of these treatments have been confirmed by clinical studies, little is known about the effectiveness of ICI treatment. Although investigators have identified various markers associated with the immunotherapy response, such as TMB, TIC, and immune gene signatures, the application of immunotherapy has been limited due to limitations and differences between studies (Gibney et al., 2016; Chan et al., 2019; Samstein et al., 2019).

In studies of BLCA, increasing attention has been paid to infiltrating immune cells (Jing et al., 2022), especially eosinophils. It is well known that eosinophils have been shown to infiltrate multiple tumors and are able to regulate tumor progression either directly by interacting with tumor cells or indirectly by shaping TIME. Previous reports elucidate the complex interactions between different immune cell types in TIME and the effects on the prognosis of cancer patients (Varn et al., 2017). The pharmacological blocker of the eosinophilic immune checkpoint is an approach for cancer immunotherapy. These molecules primarily control T-cell activity, including cytotoxic T-lymphocyte-associated protein4, PD1, and PDL1 (Pardoll, 2012; Webster, 2014).

In the current work, we collected two separated cohorts from TCGA-BLCA and GSE31684 to explore the potential roles of eosinophil-related genes in BLCA patients. 414 tumor samples and 16,394 associated genes were used for further studies. Twenty-two tumor-infiltrating immune cell subsets were obtained using the CIBERSORT algorithm. Next, we identified a module (greenyellow) that was positively associated with eosinophil-related genes using the WGCNA approach, which contained 313 candidate genes. After a series of screening, nine eosinophil-related genes (*AGXT, B3GALT2, CCDC62, CLEC1B, CLEC2D, CYP19A1, DNM3, SLC5A9, SLC26A8*) were finally identified. RS are then calculated and prognostic features are constructed. Risk characteristics were considered independent prognostic predictors of BLCA and performed well in univariate and multivariate regression analyses.

Using known samples, we constructed a nomogram based on RS and other clinical data, including gender, tumor grade, clinical stage, T, N, and M stage. It reduces statistical prediction models to a single numerical estimate of 1-, 3-, and 5-years OS tailored to individual patient conditions. These predictions of individual clinical outcomes can provide valuable information for clinical decision-making. The nomogram transforms the complex regression formula into a visual graph, making the results of the prediction model more readable and more valuable (Iasonos et al., 2008; Wang et al., 2022). Therefore, it is suggested that nomogram can be more widely concerned and applied in future medical research and clinical practice.

Studies have found a correlation between genetic alternation and immunotherapy response (Burr et al., 2017; George et al., 2017). Our results showed that TMB, a predictor of immunotherapy sensitivity, increased significantly with RS. By plotting the stratified survival curves, we found that RS had prognostic predictive power independent of TMB, which also confirmed that TMB and RS represent different aspects of immunobiology. In addition, our study also found significant differences between HRG and LRG gene variant frequencies and transcriptome levels.

The PPAR signaling pathway of LRG was activated in GSVA, while HRG was associated with *WNT, TGF-BETA, MAPK, and CHEMOKINE* signaling pathways. These results suggest that there exist significant differences in the molecular mechanisms between the high and low risk groups. In addition, risk scoring schemes showed that chemotherapeutic drug sensitivity was associated with RS. Based on the above results, we speculated BLCA patients may be better suited for different combinations of molecularly targeted and chemotherapeutic agents based on risk stratification.

In addition, RS was significantly and positively correlated with ICI-related gene CTLA4, indicating that high-risk samples might be more affected by immune checkpoint blockade. In the IPS risk scoring system, there were high IPS scores in the HRG patients (except PD-1/PD-L1/PD-L2 positivity and CTLA-4 positivity), suggesting low-risk patients are more suitable for novel ICB-targeted therapy, whereas not PD1/CTLA4 combination immunotherapy.

Through the previous RS formula, it is not difficult to find that the expression of *SLC5A9* and *CLEC2D* genes is negatively correlated with the RS. Interestingly, however, we found that *SLC5A9* and *CLEC2D* genes had opposite associations with immune checkpoint-related genes (Figure 10B). *CLEC2D* gene was positively correlated with most immune checkpoint-related genes, while *SLC5A9* gene was negatively correlated with most immune checkpoint-related genes. This may seem contradictory, but it is not. The impact of eosinophil-associated genes on the risk score is comprehensive and comprehensive, and its impact on the prognosis of BLAC includes tumor occurrence and progression, as well as the therapeutic effects of chemotherapy, surgery, and immunotherapy. Therefore, it is understandable that there are differences in the effects of *SLC5A9* and *CLEC2D* genes on the effect of immunotherapy. Nowadays, human research on tumor progression and therapeutic effects has only taken a small step, and there are too many unknowns waiting to be explored and discovered by human beings.

Among the nine eosinophil-related genes we screened, *DNM3* gene plays an important role in tumor biological progression and clinical prognosis. Ma et al. found that the *DNM3* gene can inhibit the proliferation and metastasis of colon cancer cells through the *AKT* signaling pathway (Ma et al., 2019). Cheng and his colleagues found that the expression level of the *DNM3* gene in colorectal cancer was significantly correlated with tumor size and clinical case type (Cheng et al., 2022). Zhang et al. found that *DNM3* can attenuate the growth of hepatocellular carcinoma by activating p53 (Zhang et al., 2016). Jing Fa found that *DNM3* overexpression could inhibit the proliferation, migration, and invasion of cervical cancer cells (Fa, 2021). The specific mechanism of *DNM3* gene in BLCA in tumor biological progression and clinical prognosis is still unclear. This study is expected to have a certain reference value for *DNM3* gene tumor research.

In addition to DNM3 gene, the research of other 8 eosinophil-related genes in the tumor also made gratifying progress in recent years. The *CCDC62* gene plays a role in tumor progression of gastric (Domae et al., 2009) and prostate cancers (Chen et al., 2009). *CLEC1B* gene is associated with pancreatic cancer cachexia (Narasimhan et al., 2020). Overexpression of the *CLEC2D* gene in prostate cancer affects the biological process of the tumor (Mathew et al., 2016; Buller et al., 2020). The *CYP19A1* gene is associated with breast (Wu et al., 2017; Lv et al., 2021) and lung cancer (Shi et al., 2008). This paper corroborates the biological roles of these genes in tumors in the above studies, and deepens the understanding of the biological roles of eosinophil-related genes in BCLA.

In short, this study identifies potential directions for research related to BLCA tumor progression and clinical prognosis. Under the general trend of early diagnosis and precision medicine in the future, the results of this study are expected to provide certain guidance for individualized treatment.

## Conclusion

Overall, the distinction between eosinophil-based risk scoring schemes helps predict clinical outcomes, genetic mutations, TIME heterogeneity, and treatment response. Nonetheless, these findings require further experimental and clinical validation in different centers and larger cohorts.

## Data Availability

The original contributions presented in the study are included in the article/Supplementary Material, further inquiries can be directed to the corresponding authors.

## Abbreviations

BLCA, bladder urothelial carcinoma; TIME, tumor immune microenvironment; WGCNA, weighted gene co-expression network analysis; RS, risk score; HRG, high-risk group; LRG, low-risk group; ROC, receiver operating characteristic; TMB, tumor mutational burden; IPS, Immune Phenomenon Score; IC50s, Half-maximal inhibitory concentrations; ICI, immune checkpoint inhibitors; MIBC, muscle-invasive bladder cancer; NMIBC, non-muscle invasive bladder cancer; ORR, objective response rate; CRR, complete response rate; TCGA-BLCA, The Cancer Genome Atlas-Bladder urothelial carcinoma; TCGA, The Cancer Genome Atlas; TIC, tumor-infiltrating immune cell; OS, overall survival; GSVA, Applied Gene Set Variation Analysis; IPS, Immune Phenomenon Score; GDSC, Genomics of Cancer Drug Sensitivity; SMG, Significantly mutated gene.
